# Patient-Specific Network for Personalized Breast Cancer Therapy with Multi-Omics Data

**DOI:** 10.3390/e23020225

**Published:** 2021-02-11

**Authors:** Claudia Cava, Soudabeh Sabetian, Isabella Castiglioni

**Affiliations:** 1 Institute of Molecular Bioimaging and Physiology, National Research Council (IBFM-CNR), Via F.Cervi 93, Segrate, 20090 Milan, Italy; 2 Infertility Research Center, Shiraz University of Medical Sciences, Shiraz, Iran; soudabehsabet@gmail.com; 3 Department of Physics “Giuseppe Occhialini”, University of Milan-Bicocca Piazza dell’Ateneo Nuovo, 20126 Milan, Italy; isabella.castiglioni@unimib.it

**Keywords:** protein network, bioinformatics, breast cancer, copy number alteration

## Abstract

The development of new computational approaches that are able to design the correct personalized drugs is the crucial therapeutic issue in cancer research. However, tumor heterogeneity is the main obstacle to developing patient-specific single drugs or combinations of drugs that already exist in clinics. In this study, we developed a computational approach that integrates copy number alteration, gene expression, and a protein interaction network of 73 basal breast cancer samples. 2509 prognostic genes harboring a copy number alteration were identified using survival analysis, and a protein–protein interaction network considering the direct interactions was created. Each patient was described by a specific combination of seven altered hub proteins that fully characterize the 73 basal breast cancer patients. We suggested the optimal combination therapy for each patient considering drug–protein interactions. Our approach is able to confirm well-known cancer related genes and suggest novel potential drug target genes. In conclusion, we presented a new computational approach in breast cancer to deal with the intra-tumor heterogeneity towards personalized cancer therapy.

## 1. Introduction

For many years, personalized treatments have been primarily focused on gene sequencing or gene expression patterns in order to target specific patients. Many of these computational methods based on high-throughput data have been developed on identifying a single marker that will be quantified to determine the optimal treatment [1]. Indeed, genomic, and transcriptomic alterations are the main causes of tumorigenesis and evolution of cancer [2,3,4].

One example of precision medicine is the drug trastuzumab (Herceptin) against breast cancer. It works only for tumors that show human epidermal growth factor receptor 2 (HER2)-overexpression and/or -amplification [5]. A second example is the mercaptopurine (Purinethol) used in acute lymphoblastic leukemia. Patients with specific variants that interfere with the processing of the drug should perform a genetic testing in order to direct the patient to the right therapy [5].

However, several studies demonstrated that a protein could have either a pro or an anti-apoptotic effect, depending on the signaling network context [6]. The study of the signaling network has direct implications on biomarker discovery. Indeed, several studies reported a shift from the quantification of biomarkers to the estimation of signaling networks [7].

Protein interaction networks present gene products that physically interact with each other to achieve specific cellular functions, such as metabolism, cell cycle control, and signal transduction [8]. These signaling networks have been proven to be particularly crucial for the study of the relationships between network structures and function [9], and for the identification of novel protein function [10]. In addition, protein interaction networks have become necessary tools for correlating proteins with distinct phenotypes and diseases [11], as well as for investigating pharmacological drug-target associations [12]. The alterations of protein interaction networks caused by a disease, may vary significantly among patients due to patient-specific mutations. The networks represent how the altered proteins are connected. Furthermore, the same protein can be altered in different patients, but it can participate in different networks, due to patient-specific network reorganization [13].

Important prior information that has been largely ignored in protein interaction studies is the relationship between gene expression profiles and recurrent disease or patient’s survival. The integration of survival models with protein interaction networks is expected to achieve more accurate prognoses or diagnoses [14]. A recent pan-cancer study focused on transcriptome and clinical data, reported that protein interactome alterations among different cancer types had an impact on the clinical outcomes [15].

Genomic alterations can modify biological networks such as protein–protein interactions influencing the overall survival of the patient. Therefore, investigation of the effects of mutations/alterations could offer personalized drug treatments [16].

The need to predict the effects of a drug on the individual patient led to the development of different computational methods [17,18]. One example integrating genomic and transcriptomic profiles is HIT’nDRIVE. It is able to reveal patient-specific genes that can influence the expression of transcripts. The method applied to 2200 tumors identified driver genes associated with survival outcomes, and predicted drug efficiency [19].

Mateo et al. presented a computational method to investigate driver alteration co-occurrence patterns in patient-derived mouse xenograft. They explored the molecular profiles of responder and non-responder samples to specific drugs and they found biological networks that are able to predict dug efficacy [20].

A recent study by Chierici et al. proposed a network-based framework, Integrative Network Fusion (INF), to identify multi-omics predictive biomarkers. It is based on machine learning models and was tested on three different datasets originated from The Cancer Genome Atlas [21].

PanDrugs is another computational framework that studies gene-drug interactions integrating genomic profiles, biological pathway and pharmacological evidence to address the patients through a personalized therapy [22].

However, despite the development of these novel computational approaches, only 28 genes were approved as biomarkers by the Food and Drug Administration (FDA) [23].

Breast cancer (BC) is the most frequent tumor in women worldwide. BC is diagnosed with four subtypes: luminal A, luminal B, Her2 amplified, and basal like. Basal-like BC, which lacks estrogen, progesterone receptor, and HER2, is an aggressive BC subtype with limited treatment strategies, mainly due to the phenomena of drug resistance. Therefore, identification of new drug targets to improve current treatment strategies and to overcome these phenomena is a major goal [24].

Here, we present a computational strategy to map the patient-specific protein interaction networks for basal BC patients that emerge combining prognostic and altered genes obtained from The Center Genome Atlas (TCGA) dataset. We selected each patient with genes that show a DNA alteration and have a prognostic role demonstrated with a survival analysis. Then, we generated a patient-specific network by considering the direct protein–protein interactions between altered and prognostic genes. We selected, for each patient-specific network, the top five genes with a higher degree centrality and proposed know drugs that could be effective in BC therapy. We validated the top five genes of protein networks using two independent datasets of copy number profiles from the Gene Expression Omnibus (GEO).

## 2. Materials and Methods

### 2.1. Data

Copy number and gene expression dataset of 73 basal BC, as well as overall survival, were obtained from The Center Genome Atlas (TCGA) database. The download and pre-processing steps for copy number and transcriptomic data were performed using the TCGABiolinks package [25].

Copy number profiles of the basal BC samples from TCGA were downloaded using the getGistic function with type = ‘thresholded’ of TCGABiolinks R package. The copy number matrix contains altered genes for each patient. It includes integer values from −2 to 2: ±2 represents strong amplification/deletion, ±1 mild amplification/deletion, and 0 means no alterations detected. We considered genes with positive and negative numbers that show amplification and deletion, respectively.

In the validation step, copy number profiles for independent datasets from GEO dataset (GSE87048 and GSE26232) were calculated using the package aroma.affymetrix with CRMAv2, Circular Binary Segmentation model, and GISTIC tool. We considered eight basal BC samples from GSE87048 and 17 basal BC samples from GSE26232.

BC molecular subtypes had previously been identified with PAM-50 classification [26].

The interaction between drug and protein was obtained using DGIdb database [27], Matador database [28], and pharmagkb [29]. Approved drugs in cancer by Food and Drug Administration (FDA) are considered (National Cancer Institute, 2018). The association between drugs and BC was obtained using 69 drugs to treat BC already known and approved by FDA [https://www.cancer.gov/about-cancer/treatment/drugs/breast].

### 2.2. Survival Analysis

The Kaplan-Meier method was applied to generate survival curves using the transcriptomic dataset of basal BC. We performed the survival analysis on the 73 basal BC samples from TCGA database, and the differences between survival curves were evaluated using the log-rank test [30,31]. We used the R-package survival for survival analyses (v.3.1–11) [30].

The cut-off points for the high or low-expression group for each gene were changed by iteratively selecting the genes and cut-off that stratify the two groups, minimizing the *p*-values. *p*-values were corrected using the Benjamini-Hochberg procedure for multiple testing adjustments [32]. Adjusted *p*-values less than 0.01 were considered significant. The cut-off was selected to obtain more than 5% of the samples for each group [33].

### 2.3. Protein-Protein Interaction

We used SpidermiR to download protein–protein interactions that contain interactions derived from different public databases (for details, see Reference [34]). We created a protein network for each sample, considering the direct interactions of altered genes with a prognostic role. We found the genes with a central role in the network for each network. The central role of a gene in a network can be quantified by the degree centrality. Specifically, the degree centrality of a gene indicates the number of its connections. The nodes with a higher degree centrality are the most essential. Thus, we determined the top five genes with a higher degree centrality for each protein–protein interaction network.

## 3. Results

### 3.1. Proposed Approach

Our aim was to discover prognostic gene expression profiles in basal BC that may be associated with copy number alterations. The algorithm is based on the survival analysis of gene expression profiles to select prognostic genes and then integrating copy number alterations information, we created patient-specific interaction network.

**Figure 1:**
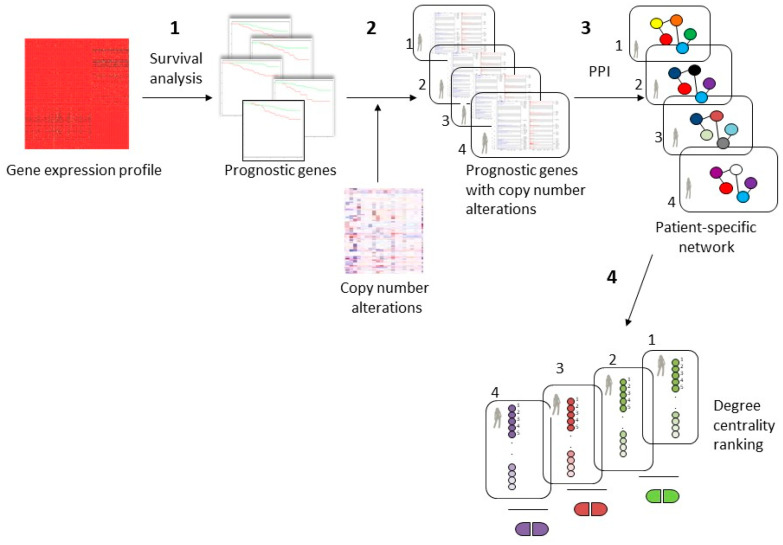
Workflow of the proposed approach for personalized cancer therapy. (1) A dataset of gene expression profile of basal breast cancer was examined with a survival analysis identifying prognostic genes; (2) identification of subsets of prognostic genes that have copy number alterations for each patient; (3) patient-specific protein-protein interaction (PPI) network considering the direct interaction among prognostic genes with copy number alterations; (4) patient-specific drugs revealed using the top 5 genes with the higher degree centrality in the protein–protein interaction network.

The proposed computational approach presented in Figure 1 consists of four steps.

In the first step, we performed a survival analysis using gene expression profiles of 73 basal BC samples from TCGA, and we identified the prognostic genes that are able to predict good and poor prognosis groups.

In the second step, we identified, for each patient, the genes that exhibited a copy number alteration and that had a prognostic role. Different subset of altered genes can originate from this step since copy number alterations can be different for each patient.

In the third step, we generated different protein–protein interaction networks for each patient considering the direct interactions among the prognostic genes with copy number alteration.

Finally, in the fourth step, we revealed the top five genes, using degree centrality, with a crucial role in the patient-specific network. Then, we analyzed the known drug-gene interactions, and we identified some drugs that could regulate the genes with a central role in the patient-specific network.

### 3.2. Step 1: Survival Analysis

In the first step, we performed a survival analysis on a transcriptomic dataset of 73 basal BC samples from TCGA data. We found that 2748 genes have a prognostic role since they can classify the samples with good and poor prognosis.

### 3.3. Step 2: Copy Number Analysis

In the second step, we analyzed the copy number alterations for each of the 73 basal BC samples. The aim was to identify copy number alterations that may be associated with prognostic gene expression profiles in basal BC. We selected prognostic genes that also harbor a copy number aberrant event and we obtained that 2509 out of 2748 genes have a copy number alteration in at least one sample.

### 3.4. Step 3: Protein-Protein Interaction Analysis

We constructed a protein–protein interaction network for each patient involving the 2509 genes. Different networks among patients can represent how the different altered genes are connected. Only direct interactions between altered and prognostic genes were considered.

**Figure 2:**
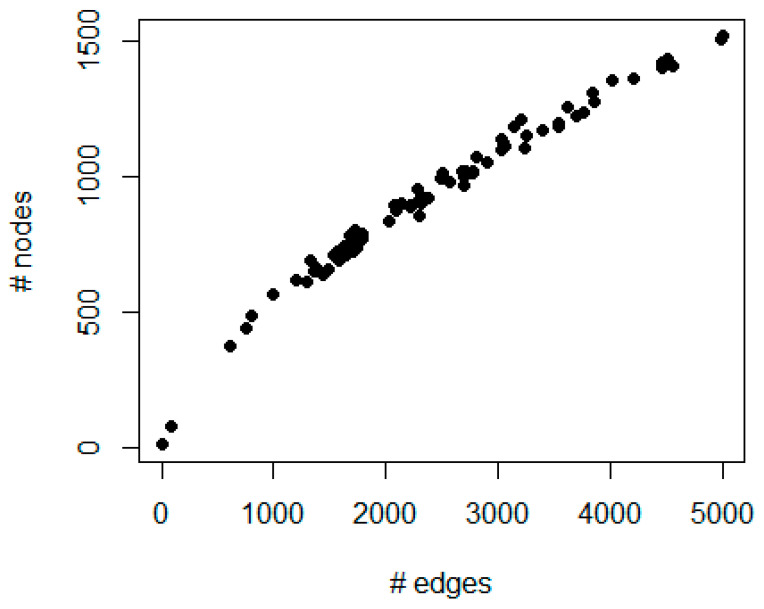
The figure represents a scatter plot of the number of edges (*x*-axis) and nodes (*y*-axis) for each patient in the protein–protein interaction network generated by integrating prognostic genes and copy number alterations.

Figure 2 shows a scatter plot of the number of edges and nodes for all 73 basal BC samples. The number of edges ranges from 4 to 4999; the number of nodes ranges from 8 to 1514.

### 3.5. Step 4: Drug-Protein Interaction Analysis

In the fourth step, we integrated the drug-gene interactions into patient-specific networks. Overall, we found that 91 out of 2509 genes are targets of 42 out of 69 FDA approved drugs. We calculated the degree centrality for each network, and we selected the top five genes with the highest degree centrality. We chose the only known drugs that interact (if present) with the top five genes. The patient-specific network of altered genes provides the basis for personalized drug design. A proper treatment would be chosen based on the discovered targetable gene biomarkers and the available FDA-approved drugs.

We found that only 18 combinations of seven crucial genes (with a known drug-gene interaction) describe the entire dataset characterizing the inter-tumor heterogeneity of basal BC. 18 groups of combined genes, consisted of seven altered and prognostic genes (BRCA1, HDAC2, MCL1, PIK3R1, PSMD4, RB1, and TP53), describe the biological alterations that differentiate the 73 basal BC patients of TCGA dataset (Table 1).

Forty out of 73 basal BC patients (55% of samples) show a simultaneous alteration of BRCA1 and TP53. Indeed, in these samples, both genes have a central role in the network, concluding to be prognostic genes with a copy number alteration. Various drug combinations were suggested (Table 1). For example, six drugs (cyclophosphamide, doxorubicin, gemcitabine, paclitaxel, olaparib, and tamoxifen) act on both genes. In clinics, basal BC patients harboring an alteration of networks involving BRCA1 and TP53 would be suggested by the BRCA1/TP53- targeting drugs.

Surprisingly, the survival analysis reported that high expression of BRCA1 is associated with a poor prognosis. In the basal BC cohort, we obtained 66 samples with low expression of BRCA1 and seven samples with high expression. This result could be consistent with the absence of evidence that the down-regulation of BRCA1 contributes to sporadic BC. A previous study hypothesized that low BRCA1 expression may describe a small subgroup of basal BC, whose molecular mechanisms are not yet clear. Another possible scenario is the presence of other factors that influence the expression level of BRCA1 such as epigenetic regulation [35]. Indeed, we found that four out of seven samples with high expression of BRCA1 present deletions of gene and three samples with no alteration in the gene. Overall, copy number analysis confirmed that the most present DNA alteration is the deletion in 44 out of 73 samples, 21 samples did not show alterations, and 8 amplifications of BRCA1. For the drug analysis, we found that seven samples with high expressions belong to the predominant group reported in Table 1. Concerning the therapy of the poor prognosis group with a higher expression of BRCA1 (7 samples) we could predict a poor response to chemotherapy since previous studies demonstrated that patients with high levels of BRCA1 could present a lower response to chemotherapy with respect to BRCA1 mutated patients [36,37]. Among the 40 BC samples that had an important role in BRCA1 and TP53: 34 samples showed a deletion and six had an amplification.

Regarding TP53, the survival analysis reported that its high expression is associated with a poor prognosis. In basal BC cohort we obtained 68 samples with low expressions of TP53 and five samples with high expression. This result seems to be consistent with previous studies [38,39]. We found that three out of five samples with high expression of TP53 present deletions of gene and two samples had no alteration in the gene. Like BRCA1, copy number analysis performed on TP53 reported that the most present DNA alteration is the deletion in 50 out of 73 samples, 18 samples did not show alterations and five amplifications of TP53. For the drug analysis, we found five samples with high expressions of TP53 belong to the predominant group reported in Table 1. Among 40 BC samples that had an important role of BRCA1 and TP53: 90% of samples present a deletion in TP53 and 10% amplification.

Networks with a principal alteration of TP53 were identified in 7 out of 73 patients suggesting one of 12 drugs (gemcitabine, epirubicin, doxorubicin, paclitaxel or capecitabine, docetaxel, thiotepa, abemaciclib, tamoxifen, olaparib, cyclophosphamide, or alpelisib) as personalized cancer therapy regimen. All these drugs regulate TP53. All seven samples belonging to this group harbor a deletion of TP53 and 85% of samples belongs to the group have a low expression of TP53 according to the survival analysis.

Six out of 73 patients showed a principal alteration of PSMD4 with a central role in the network. Among FDA-approved drugs, talazoparib was experimentally validated and act on PSMD4. The survival analysis revealed that a high expression of PSMD4 is correlated with a poor prognosis. In the basal BC cohort, we identified 61 samples with a low expression of BRCA1 and 12 samples with high expression. We found that nine out of 12 samples with high expression of PSMD4 present deletions of gene and three samples had no alteration in the gene. However, copy number analysis reported that the most present DNA alteration is amplification in 88% of the samples. Deletions were reported in 12% of the samples. None of the samples had no alterations in the gene.

In addition, our approach is also able to find genes that currently do not have a clear link to cancer and do not appear to be the target of approved drugs to treat BC. For example, among them we found that TRAF6, EP300, YWHAQ, HSPA8 and VIM showed a crucial role in the patient-specific network being genes with a high degree centrality.

TRAF6 was found altered in 44 out of 73 samples: 20 samples have a deletion of gene and 24 showed an amplification. In our study, high expression of TRAF6 is also associated with poor prognosis. In addition, 57% of samples with high expression of TRAF6 have an amplification of TRAF6. Our study suggested that TRAF6 inhibitors could be candidate drugs in patients with high TRAF6 expression level.

EP300 showed a deletion in 25 samples and an amplification in 21 samples. Survival analysis indicated that low expression of EP300 is also associated with poor prognosis. 33% of samples with a low expression of EP300 showed a deletion. The rest of the samples belonging to the low expression group did not present alterations in EP300. However, the low expression group only contains three samples and further analyses should be performed.

YWHAQ was found to be amplified in the majority of samples (38 samples), and deleted in 11 samples. Low expression of YWHAQ is also correlated with poor prognosis. Like EP300, the low expression group contains only three samples and further analyses should be performed.

Twenty-nine out of 73 samples showed no alteration in HSPA8, while 27 samples reported a deletion and 17 reported amplification. In our study we reported that high expression of HSPA8 is also associated with poor prognosis. Copy number analysis did not show a consistency of results with survival analysis. Indeed, 22% of samples belonging to high expression group showed amplification, 55% of the samples a deletion and 22% of the samples did report any alterations.

Forty-eight samples out of 73 samples showed a amplification and 5 a deletion in VIM. Low expression of VIM is also correlated with poor prognosis. 50% of samples with a high expression level of VIM did not report alterations in the gene.

### 3.6. Validation

We validated the results obtained using the computational approach on two independent GEO datasets: GSE87048 and GSE26232 containing 8 and 17 basal BC samples, respectively.

**Figure 3:**
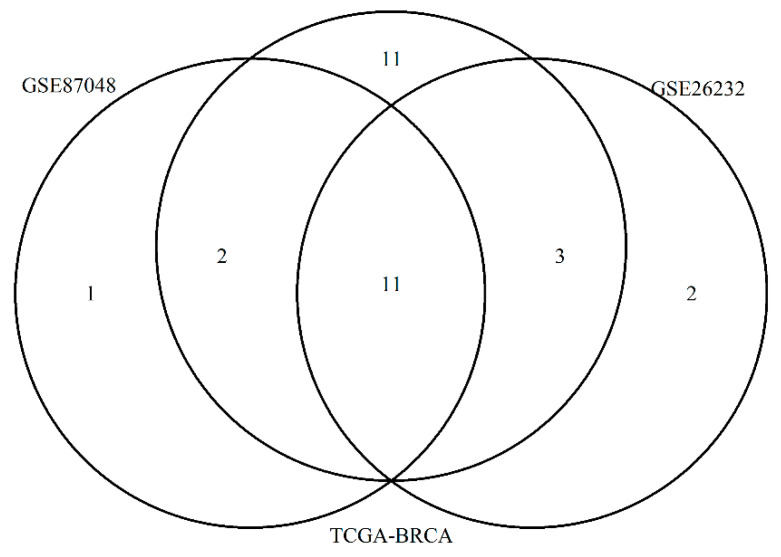
The figure represents a Venn Diagram showing the overall top 5 genes for each patient with higher degree centrality in the three different datasets: TCGA-BRCA, GSE87048 and GSE26232.

We created a patient-specific network in the two GEO datasets by considering the direct interactions between the 2748 prognostic genes if altered in that sample. We selected the top five genes with the highest degree centrality for each patient-specific network and we represented with a Venn diagram common and unique genes found in validation sets (Figure 3).

Eleven genes were found in common among three datasets: TP53, MYC, TRAF6, BRCA1, YWHAQ, EP300, HSPA8, EGR1, PSMD4, PIK3R1 and RB1.

Eleven genes were identified only in TCGA data: C18orf8, HAX1, INTS3, MCL1, YWHAG, CUL5, SNCA, HNRNPU, IKBKE, DDX5, and RPA3.

One gene ACACA were found only in GSE87048, and 2 genes in GSE26232: ACTB and CDK9.

We found that one of the main patterns in GSE87048 is BRCA and TP53, the same found in 40 of the 73 TCGA samples (Table 2). The other main patterns are BRCA1-HDAC2 and BRCA1-PSMD4.

We validated the computational procedure using another independent GEO dataset: GSE26232. It contains 17 basal BC samples. We processed the data, and we obtained the altered patterns, as reported in Table 3. We found that the main altered pattern consists of BRCA1 and TP53 (as reported in the previous datasets), followed by BRCA1 and PSMD4.

## 4. Discussion

In this study, we proposed a computational approach to accurately map cancer patients according to their basal BC-specific molecular aberrations. For this purpose, we implemented a survival analysis of 73 basal BC patients from TCGA, selecting the genes with a prognostic role. We obtained that 2748 genes out of 15,237 are prognostic genes. Furthermore, through a copy number alteration analysis, 2509 out of 2748 genes were identified as being altered in at least one patient. Since each patient harbors a specific alteration of these genes, we generated a protein interaction network evaluating the direct interactions among 2509 genes if altered for that patient. We described the individual protein network of the specific patient.

We showed that patient-specific protein interactions assigned to each patient, allow designing single drugs or combinations of drugs, which already exist in clinics. We found that the 18 combinations of seven altered proteins could fully characterize this set of basal BC patients.

Our computational approach is powerful in linking alterations at the genomic level to modifications at the transcriptome level. Therefore, we identified a limited number of altered genes that can elucidate the observed transcriptional changes. The determined genes include seven genes with a well-known role: BRCA1, HDAC2, MCL1, PIK3R1, PSMD4, RB1, and TP53.

BRCA1, a tumor suppressor gene, which is commonly altered in familial BC, was found as the first biomarker able to predict the risk of BC development. The role of BRCA1 is wide ranging from DNA damage repair and chromatin remodeling to regulating apoptosis and cell cycle [40].

HDAC2 is a histone deacetylase that plays a role in DNA repair and immune function. However, the knowledge of the genomic and transcriptomic alterations of HDAC2 is limited. A recent study integrating copy number alteration, gene expression, and survival analysis revealed that the up-regulation of HDAC2 is significantly correlated with poor prognosis. This study suggested that HDAC inhibitors could be a promising new group of anticancer agents [41].

MCL1 belongs to the BCL2 family and is a negative modulator of apoptosis. Amplifications of MCL1 are observed in several human cancers such as breast, lung, and ovarian cancers [42]. A recent study suggested that MCL1 inhibitors could have a role in estrogen receptor-positive BC blocking cell survival [43].

PIK3R1, a tumor suppressor gene, belongs to PI3K pathway that is one of the most altered pathways in cancer. Several studies reported a down-regulation of PIK3R1 in many human cancers. A previous study provided evidence that the alterations of PIK3R1 could be an indicator of sensitivity to trametinib or MEK inhibitors in BC treatment [44].

The PSMD4 encodes ubiquitin-binding protein, and angiocidin. These proteins have a significant role in tumor progression: ubiquitin-binding protein plays a role in the cellular cycle progression, and angiocidin is antiangiogenic, which inhibits tumor development. Previous studies have speculated a molecular mechanism of PSMD4 involving inhibition of BC proliferation via epidermal activation growth factor receptors [45].

Retinoblastoma protein (RB1) and TP53 are tumor suppressor genes, which could avoid tumoral cell growth by promoting cancer cell death. The inactivation of RB1 is frequently assessed in triple-negative BC and is linked to sensitivity to many cancer drugs, including TOP1 inhibitors [46,47].

In our study, the combination of these seven prognostic and altered genes allow us to obtain different patient-specific networks.

It was found that 55% of the TCGA samples, 25% of the GSE87048 and 35% of GSE26232 have as one of the main altered patterns BRCA1 and TP53. Our study suggests that the potential drugs for these patients could be cyclophosphamide, doxorubicin, gemcitabine, olaparib, paclitaxel, or tamoxifen.

Previous studies reported that many triple-negative BC patients that harbor mutations in BRCA1 have also TP53 mutations: these findings showed that TP53 mutations are a requisite for BRCA1 alterations. This provides some evidences that there is a dependency on the loss of TP53 in BRCA altered human BC [48]. Interestingly, Manié et al. demonstrated that TP53 mutations occur in almost all sporadic basal BC samples (92%) while only 53% of luminal BC samples show a recurrent TP53 mutation [49]. Among 6 drugs proposed in these samples, PARP inhibitor, olaparib has been recently approved in the therapy of BRCA1 altered BC samples [50].

10% of TCGA samples and 12% of GSE26232 samples present the alteration of TP53. The use for these patients of gemcitabine, epirubicin, doxorubicin, paclitaxel, capecitabine, docetaxel, thiotepa, abemaciclib, tamoxifen, olaparib, cyclophosphamide or alpelisib, acting on TP53, was suggested by this study.

4% of the TCGA samples, 25% of the GSE87048 samples and 18% of GSE26232 samples showed an alteration of BRCA1 and PSMD4. Talazoparib could be a personalized drug for those patients that show this altered pattern. Indeed, our study confirms a previous study that cell lines with copy number alterations of PSMD4 are significantly more sensitive to talazoparib [51].

Our approach was also able to find new potential drug-targets of BC: TRAF6, EP300, YWHAQ, HSPA8 and VIM. Currently, these genes appear to not be targets of drugs used for BC.

TRAF6, a ubiquitin ligase, is associated to AKT generating its multi-ubiquitination. TRAF6 is also involved in the activation of mTROC1 and PI13K [52]. While little is known regarding the association of TRAF6 in breast cancer, it was found over-expressed in triple negative BC and it expression correlates with metastatic outcome [53]. In addition, a recent study reported that a molecule, an inhibitor of TRAF6, decreases metastasis in mouse BC models [54].

EP300 is described as oncogene in triple negative BC regulating cancer stem cell. A knockdown of EP300 decreased the expression of ABCG2, a gene involved in drug resistance [55]. Other studies showed that EP300 increases migration and the invasiveness of cancer cells [56].

YWHAQ is a gene involved in apoptosis, regulating TP53 through post-translational modifications [57]. YWHAQ was found to be up-regulated along with nine other genes in BC patients compared to normal samples [58]. Considering these studies with our results YWHAQ could be a potential new drug target.

HSPA8 is located on a region deleted in 40% of sporadic BC, the chromosome 11q23.3 [59]. It plays a role in protein folding and differentiation. A previous study examined HSPA8 1541–1542delGT polymorphism in BC that can vary HSPA8 protein expression [60]. A recent study in multiple human cancers proposed HSPA8 as potential exome markers for cancer detection. Indeed, it was the only protein identified in >50% of extracellular vesicles and particles samples [61].

VIM is a mesenchymal biomarker expressed in different types of tissues during differentiation processes and tumor evolution. It is used as a marker of epithelial-mesenchymal transition and its up-regulation is associated with lymph node involvement, higher disease stages as well as poor prognosis [62].

In conclusion, we suggest that BC can be characterized by distinct network alterations in order to develop a novel patient-specific treatment.

## 5. Conclusions

In this study, we presented a computational approach, integrating copy number alteration, gene expression, and survival analysis of basal BC samples.

We identified 2748 prognostic genes with survival analysis using gene expression profiles of 73 basal BC from TCGA data. Furthermore, we analyzed the copy number expression profiles of the same TCGA samples, and we selected the altered genes that were also able to classify the samples with good and poor prognosis, obtaining 2509 out of 2748 genes. Through a network analysis, we found seven crucial genes (BRCA1, HDAC2, MCL1, PIK3R1, PSMD4, RB1, and TP53) with known drug-gene interactions that can characterize the 73 basal BC. In addition, our approach can find also novel drug-targets such as TRAF6, EP300, YWHAQ, HSPA8 and VIM that showed a central role in the protein–protein interaction network.

The current method is able to identify patient-specific protein interactions that could address the patients on the use of single drugs or combinations of drugs, which already exist in clinics.

However, our study has some limitations. Gene expression is affected by other factors including DNA mutations and epigenetic events such as DNA methylation, non-coding RNA and histone modifications. These events should be included in future research. Furthermore, the current findings were obtained with a computational approach and future studies should be performed with a validation in the laboratory.

## Figures and Tables

**Table 1:** Prediction of patient-specific therapy for 73 basal breast cancer from The Cancer Genome Atlas.

Proteins	Frequency	Drugs
BRCA1, TP53	40	cyclophosphamide or doxorubicin or gemcitabine or olaparib or paclitaxel or tamoxifen (BRCA1 and TP53)
TP53	7	Gemcitabine or epirubicin or doxorubicin or paclitaxel or capecitabine or docetaxel or thiotepa or abemaciclib or tamoxifen or olaparib or cyclophosphamide or alpelisib (TP53)
PSMD4	6	Talazoparib
BRCA1, PSMD4	3	talazoparib (BRCA1 and PSMD4)
BRCA1, HDAC2, TP53	2	olaparib (BRCA1, HDAC2 and TP53)
BRCA1, PSMD4, TP53	2	talazoparib (BRCA1 and PSMD4) and gemcitabine or epirubicin or doxorubicin or paclitaxel or capcitabine or docetaxel or thiotepa or abemaciclib or tamoxifen or olaparib or cyclophosphamide or alpelisib (TP53)
PSMD4, RB1	2	talazoparib (PSMD4) and fulvestrant or palbociclib or methotrexate or ribociclib or everolimus (RB1)
BRCA1	1	doxorubicin or cyclophosphamide or talazoparib or olaparib or gemcitabine or tamoxifen or paclitaxel or everolimus (BRCA1)
BRCA1, PIK3R1	1	doxorubicin or cyclophosphamide or talazoparib or olaparib or gemcitabine or tamoxifen or paclitaxel or everolimus (BRCA1) and alpelisib (PIK3R1)
BRCA1, PIK3R1, PSMD4, TP53	1	talazoparib (BRCA1 and PSMD4) and alpelisib (PIK3R1 and TP53)
BRCA1, PIK3R1, TP53	1	cyclophosphamide or doxorubicin or gemcitabine or olaparib or paclitaxel or tamoxifen (BRCA and TP53) and alpelisib (PIK3R1)
BRCA1, RB1	1	everolimus (BRCA1 and RB1)
HDAC2	1	olaparib (HDAC2)
HDAC2 PIK3R1 TP53	1	olaparib (HDAC2 and TP53) alpelisib (PIK3R1)
MCL1	1	docetaxel (MCL1)
PSMD4, TP53	1	talazoparib (PSMD4) and gemcitabine or epirubicin or doxorubicin or paclitaxel or capecitabine or docetaxel or thiotepa or abemaciclib or tamoxifen or olaparib or cyclophosphamide or alpelisib (TP53)
RB1	1	fulvestrant or palbociclib or methotrexate or ribociclib or everolimus
RB1, TP53	1	fulvestrant or palbociclib or methotrexate or ribociclib or everolimus (RB1) and gemcitabine or epirubicin or doxorubicin or paclitaxel or capecitabine or docetaxel or thiotepa or abemaciclib or tamoxifen or olaparib or cyclophosphamide or alpelisib (TP53)

**Table 2:** Prediction of patient-specific therapy for 8 basal breast cancers from GSE87048.

Proteins	Frequency	Drugs
BRCA1 TP53	2	cyclophosphamide or doxorubicin or gemcitabine or olaparib or paclitaxel or tamoxifen (BRCA1 and TP53)
BRCA1 HDAC2	2	olaparib (BRCA1 and HDAC2)
BRCA1 PSMD4	2	talazoparib (BRCA1 and PSMD4)
BRCA1 RB1 TP53	1	cyclophosphamide or doxorubicin or gemcitabine or olaparib or paclitaxel or tamoxifen (BRCA1 and TP53) and fulvestrant or palbociclib or methotrexate or ribociclib or everolimus (RB1)
HDAC2 PIK3R1 PSMD4	1	olaparib (HDAC2) alpelisib (PIK3R1) talazoparib (PSMD4)

**Table 3:** Prediction of patient-specific therapy for 17 basal breast cancers from GSE26232.

Proteins	Frequency	Drugs
BRCA1 TP53	6	cyclophosphamide or doxorubicin or gemcitabine or olaparib or paclitaxel or tamoxifen (BRCA1 and TP53)
BRCA1 PSMD4	3	talazoparib (BRCA1 and PSMD4)
BRCA1	2	doxorubicin or cyclophosphamide or talazoparib or olaparib or gemcitabine or tamoxifen or paclitaxel or everolimus (BRCA1)
TP53	2	Gemcitabine or epirubicin or doxorubicin or paclitaxel or capecitabine or docetaxel or thiotepa or abemaciclib or tamoxifen or olaparib or cyclophosphamide or alpelisib (TP53)
ACTB PSMD4 TP53	1	Cyclophosphamide (ACTB and TP53) and talazoparib (PSMD4)
BRCA1 PSMD4 RB1 TP53	1	cyclophosphamide or doxorubicin or gemcitabine or olaparib or paclitaxel or tamoxifen (BRCA1 and TP53) and Talazoparib (PSMD4) and Gemcitabine or epirubicin or doxorubicin or paclitaxel or capecitabine or docetaxel or thiotepa or abemaciclib or tamoxifen or olaparib or cyclophosphamide or alpelisib (TP53)
PIK3R1	1	Alpelisib
PSMD4 TP53	1	talazoparib (PSMD4) and gemcitabine or epirubicin or doxorubicin or paclitaxel or capecitabine or docetaxel or thiotepa or abemaciclib or tamoxifen or olaparib or cyclophosphamide or alpelisib (TP53)

## Data Availability

Publicly available datasets were analyzed in this study. This data can be found here: https://portal.gdc.cancer.gov/; https://www.ncbi.nlm.nih.gov/geo/query/acc.cgi?acc=GSE87048; https://www.ncbi.nlm.nih.gov/geo/query/acc.cgi?acc=GSE26232.
